# Video-Based Identification and Prediction Techniques for Stable Vessel Trajectories in Bridge Areas

**DOI:** 10.3390/s24020372

**Published:** 2024-01-08

**Authors:** Woqin Luo, Ye Xia, Tiantao He

**Affiliations:** 1 Department of Bridge Engineering, School of Civil Engineering, Tongji University, Shanghai 200092, China; 2 Ningbo Municipal Facilities Center, Ningbo 315100, China

**Keywords:** vessel-bridge collision, vessel trajectory tracking, vessel trajectory prediction, GAT, transformer

## Abstract

In recent years, the global upswing in vessel-bridge collisions underscores the vital need for robust vessel track identification in accident prevention. Contemporary vessel trajectory identification strategies often integrate target detection with trajectory tracking algorithms, employing models like YOLO integrated with DeepSORT or Bytetrack algorithms. However, the accuracy of these methods relies on target detection outcomes and the imprecise boundary acquisition method results in erroneous vessel trajectory identification and tracking, leading to both false positives and missed detections. This paper introduces a novel vessel trajectory identification framework. The Co-tracker, a long-term sequence multi-feature-point tracking method, accurately tracks vessel trajectories by statistically calculating the translation and heading angle transformation of feature point clusters, mitigating the impact of inaccurate vessel target detection. Subsequently, vessel trajectories are predicted using a combination of Long Short-Term Memory (LSTM) and a Graph Attention Neural Network (GAT) to facilitate anomaly vessel trajectory warnings, ensuring precise predictions for vessel groups. Compared to prevalent algorithms like YOLO integrated with DeepSORT, our proposed method exhibits superior accuracy and captures crucial heading angle features. Importantly, it effectively mitigates the common issues of false positives and false negatives in detection and tracking tasks. Applied in the Three Rivers area of Ningbo, this research provides real-time vessel group trajectories and trajectory predictions. When the predicted trajectory suggests potential entry into a restricted zone, the system issues timely audiovisual warnings, enhancing real-time alert functionality. This framework markedly improves vessel traffic management efficiency, diminishes collision risks, and ensures secure navigation in multi-target and wide-area vessel scenarios.

## 1. Introduction

Vessel-bridge collision accidents have a significant impact on the safety of bridges and highways, as well as the lives, property, and socio-economic development of people. These accidents are attributed to various factors, including adverse natural environmental conditions, poor navigational conditions, vessel-related issues, negligence by vessel operators, and insufficient managerial experience. Vessel-bridge collision accidents lead to serious casualties and substantial property losses, highlighting the urgent need for research into vessel collision prevention technologies [1].

Passive collision prevention methods [2,3,4] can mitigate the damage caused by vessel-bridge collisions but cannot entirely prevent the occurrence of such collisions. This study focuses on active collision prevention methods, which significantly reduce the probability of vessel-bridge collisions by providing warnings to vessels at high risk of collision. This approach helps the vessel to correct the abnormal condition and reduces the probability of bridge collisions.

Between 2010 and 2020, numerous researchers explored and optimized the selection and placement of sensors for active collision prevention methods. In 2010, Ji [5] constructed an active collision prevention system for the Sutong Bridge using an Automatic Recognition System (AIS) and Very-High-Frequency Digital Selective Calling Terminal (VHF-DSC) devices, establishing the framework for the active collision prevention system. However, AIS devices are associated with low accuracy, a low reporting frequency, and can be manually turned off, making it challenging to achieve true “active” prevention. Chen [6] deployed visible light and infrared cameras at the Shanghai Dazhihe Highway Bridge, using the AForge.NET toolkit to achieve real-time vessel detection and tracking. These types of active vessel-bridge collision prevention system devices offer significant economic benefits compared to the expensive construction costs of AIS and VHF devices. In 2013, Ren [7] implemented active warnings by deploying lasers and fusing multiple data sources, including infrared, visible light, and laser, to construct active warnings. In contrast, in 2018, Cai [8] conducted more in-depth work by fusing image data with a Full Coherent Doppler Radar at the Foshan Jiujiang Bridge. The following year, Xia [9] successfully established a large-scale navigational bridge collision prevention system in the Ningbo Sanjiangkou Area by deploying laser-based height restriction sensors, video cameras, radar, VHF, AIS, and other equipment.

The advantages and limitations of the prevailing sensor device selection schemes are summarized in Table 1. Camera sensors offer a promising research avenue due to their higher accuracy, lower cost, and the ability to capture more target features. This characteristic has made video-based active collision prevention technology the primary focus of research over the years, with vessel target detection and tracking being the core technology within this domain.

In the early mainstream detection methods, background subtraction was commonly used, while tracking methods often involved frame differencing or optical flow. Yang [12] introduced an adaptive frame differencing (AFID) tracking method for vessel targets with varying speeds. However, such target detection and tracking methods have significant flaws, notably their sensitivity to lighting conditions and their difficulty in obtaining complete motion foreground pixels, often leading to noise interference and gaps in the results.

With the advancement of computer vision technology, the three major handcrafted feature methods (HOG, LBP, HAAR) have gained prominence in the field of object detection. Chao Dong [13] utilized directional gradient histogram units combined with Fourier bases to obtain rotation-invariant gradient descriptors and applied support vector machines to recognize vessel targets from any orientation. In the domain of maritime vessel visual tracking, Liu [14] and Chen [15] have made significant improvements in and applications of the Kernelized Correlation Filter (KCF) method. These advancements have reduced the adverse impact of factors such as weather conditions and vessel obstructions on target tracking, making a profound impact on maritime vessel visual tracking.

In recent years, with the rise of deep learning, there have been revolutionary advances in object detection and tracking tasks based on video images. Mainstream object detection methods are categorized into two classes: region proposal-based two-step algorithms and end-to-end algorithms. These algorithms are not limited to video images but are also used in remote sensing images. For example, in 2020, Dong [16] used an improved Faster-RCNN network to detect objects in high spatial resolution vessel remote sensing images, expanding the sources of image data. In the same year, Xia [9] established and used a large vessel image dataset to train the SSD model, generating a vessel target detection model suitable for complex environments. At this point, vessel object detection algorithms based on video images have met the requirements of real-time and efficiency. In the field of object tracking, an outstanding single-object tracking paper called “SiamMask” appeared at the 2019 CVPR conference. Its performance represented a significant leap in comparison to other algorithms in the single-object tracking field at the time. Xi [17] improved this network to achieve excellent vessel tracking results. However, it is evident that a single target cannot adapt to real navigation scenarios. To address this, Yang [18] optimized the anchor box initialization in the YOLOv3 algorithm and combined it with the DeepSORT [19] cascade tracking algorithm to achieve better results in vessel multi-object tracking. At this point, vessel multi-object tracking algorithms have also met the requirements of efficiency and real-time performance.

Indeed, the current widely used methods are generally capable of detecting vessel targets and tracking their movements in most common scenarios. These methods typically identify the position of detected targets in two main ways: bounding boxes and heatmaps. For heatmap methods, although they can relatively accurately determine the specific point coordinates of vessel targets and track their movements, factors like large output feature maps, the need for extensive labeled data for training, high memory consumption, and limited generalization capabilities make it challenging for heatmap methods [20] to become mainstream applications.

Bounding boxes are usually determined through the output of fully connected layers. However, the use of fully connected layers can lead to the loss of spatial information in feature maps, reducing spatial generalization capabilities. Unlike this, vessels on water can have significant variations in their orientation, so using rectangular bounding boxes to extract vessel trajectories may introduce substantial errors. While some researchers, such as Feng Zhang [21], have improved bounding box structures by obtaining rotating anchor boxes to enhance the accuracy of vessel trajectory extraction, this is still a localized improvement, and bounding box methods continue to limit the potential for further improving vessel motion accuracy. While methods like YOLO combined with algorithms similar to DeepSORT and ByteTrack [22] offer fast vessel target detection, decent tracking capabilities, and robust occlusion handling, their trajectory acquisition is relatively coarse. The effectiveness of their tracking performance is intricately tied to the stability of target detection, with potential limitations in acquiring comprehensive vessel feature information. Without extensive model training, these methods are prone to false positives and missed detections, which can result in misaligned trajectory timestamps due to missed detections and incomplete trajectories due to false positives. The accuracy of such methods may not suffice to accurately track vessel motion trajectories. In the long run, discarding bounding boxes and adopting superior tracking methods is a necessary trend to achieve precise vessel motion tracking.

Vessels are often modeled as planar rigid bodies during their voyages, involving not only translation but also rotation, with changes in size as the distance varies. Therefore, a more sophisticated approach is required to acquire vessel motion trajectories for enhanced accuracy and efficiency in vessel traffic management. In such scenarios, Meta AI has introduced a method called Co-tracker [23], which is based on Transformers and is used for motion estimation in long video sequences. This method excels in conducting the dense tracking of points in extended video sequences, adeptly addressing challenges like occlusion, dynamically incorporating new tracking points as necessary, and even facilitating reverse tracking. Its robust stability is demonstrated across diverse scenarios.

Once precise vessel motion trajectories are obtained, the further prediction of vessel trajectories contributes to the implementation of an active vessel-bridge collision prevention system’s warning function in bridge areas. Predicting trajectories for vessels requires a sophisticated analysis of the spatial interactions among multiple targets, particularly when confronted with complex navigation situations involving numerous vessel targets. The STGAT method proposed by Huang [24] takes into account the spatiotemporal interactions of multiple pedestrian trajectories and delivers excellent performance. Combining the above-mentioned methods, a high-performance trajectory identification and prediction framework has been implemented. This framework utilizes a multi-feature point vessel target tracking method for long time series, obtaining high-precision trajectory data. Long Short-Term Memory (LSTM) and Graph Attention Neural Networks (GATs) are employed to predict the historical trajectories of multiple target vessels, achieving proactive warning purposes. The advantages of this framework are as follows:

1. More accurate trajectories:

The combination of YOLO and the DeepSORT algorithm relies on bounding boxes for approximating target positions, followed by post-processing methods to obtain vessel trajectory data, thus introducing complexity to the trajectory processing procedure. Notably, the accuracy of trajectories in this method is significantly affected by the precision of the detection algorithm, making it vulnerable to issues such as missed detections and false alarms. Consequently, abnormal spikes in trajectory recognition and poor robustness can arise. In contrast, our framework excels in eliminating untracked feature points in a timely manner. Leveraging the statistical characteristics of tracked feature point groups, our approach yields more accurate trajectories, offering a notable improvement over the conventional YOLO-based method.

2. Obtaining richer features—heading angle, vessel scale:

Unlike single-target detection algorithms that rely on bounding boxes to regress target position information, our framework not only captures the heading angle of the target but also extracts valuable information regarding the target’s scale and so on.

3. No missed detections or false alarms:

Automatically designating certain feature points as invisible when tracking becomes challenging, this framework seamlessly leverages statistical information from the remaining trackable points to derive vessel target trajectories and additional features. This strategic approach effectively minimizes the occurrence of missed detections and false alarms.

## 2. Vessel Trajectory Identification Technology

### 2.1. Modeling Vessel Motion under the Camera

To extract more comprehensive feature information, such as the actual heading angle and dimensions of vessels from video images, it is necessary to simplify and streamline the method for acquiring world coordinates. From the perspective of camera surveillance, within a defined monitoring range and over a certain period of time, if there is minimal fluctuation in the water level within the navigable area, it can be considered as a constant water level. Similarly, assuming a constant cargo load during vessel navigation, the actual coordinate height along the z-axis of the vessel’s tracked point can be regarded as constant. Hence, the mapping relationship between the world coordinate $Z_{w}$ of a fixed point on the vessel and its corresponding pixel coordinate is established, where i is the track point of the vessel, and t is for any frame:

$$\rho \left(u_{i}^{t},v_{i}^{t}\right)\equiv Z_{w}$$

Therefore, the process of converting pixel coordinates to world coordinates is simplified to the task of determining the world coordinates $X_{w}$ and $Y_{w}$, given the world coordinate $Z_{w}$ of a point. With this simplification, the coordinate transformation formula is derived as follows:

The following formula (Equation (1)) describes the transformation from pixel coordinates to world coordinates:

(1) $$Z_{c}\left[\begin{array}{c} u\\ v\\ 1 \end{array}\right]=\left[\begin{array}{cc} \begin{array}{cc} f_{x} & 0\\ 0 & f_{y}\\ 0 & 0 \end{array} & \begin{array}{cc} c_{x} & 0\\ c_{y} & 0\\ 1 & 0 \end{array} \end{array}\right]\left[\begin{array}{cc} \begin{array}{cc} r_{11} & r_{12}\\ r_{21} & r_{22} \end{array} & \begin{array}{cc} r_{13} & t_{1}\\ r_{23} & t_{2} \end{array}\\ \begin{array}{cc} r_{31} & r_{32}\\ 0 & 0 \end{array} & \begin{array}{cc} r_{33} & t_{3}\\ 0 & 1 \end{array} \end{array}\right]\left[\begin{array}{c} \begin{array}{c} X_{w}\\ Y_{w} \end{array}\\ \begin{array}{c} Z_{w}\\ 1 \end{array} \end{array}\right]=K\left[\begin{array}{cc} R & T\\ \vec{0} & 1 \end{array}\right]\left[\begin{array}{c} \begin{array}{c} X_{w}\\ Y_{w} \end{array}\\ \begin{array}{c} Z_{w}\\ 1 \end{array} \end{array}\right]$$

And based on the derivation in the last line of this equation, it is easy to express the coordinates of one point in the camera coordinate system $Z_{c}$, in terms of world coordinates, as shown in the following Equation (2):

(2) $$Z_{c}=\left[\begin{array}{cccc} r_{31} & r_{32} & r_{33} & t_{3} \end{array}\right]\left[\begin{array}{c} \begin{array}{c} X_{w}\\ Y_{w} \end{array}\\ \begin{array}{c} Z_{w}\\ 1 \end{array} \end{array}\right]\;\;$$

By substituting $Z_{c}$ in the camera coordinate system, the problem of scale uncertainty introduced by $Z_{c}$ is resolved. The product of the camera intrinsic matrix and the extrinsic matrix is simplified, and this resulting 3×4 matrix is denoted as follows in Equation (3):

(3) $$\left[\begin{array}{cc} \begin{array}{cc} a_{11} & a_{12}\\ a_{21} & a_{22}\\ r_{31} & r_{32} \end{array} & \begin{array}{cc} a_{13} & a_{14}\\ a_{23} & a_{24}\\ r_{33} & t_{3} \end{array} \end{array}\right]$$

Therefore, the initial transformation equation (Equation (1)) can be transformed into the following Equation (4):

(4) $$\left[\begin{array}{cc} \begin{array}{cc} r_{31} & r_{32} \end{array} & \begin{array}{cc} r_{33} & t_{3} \end{array} \end{array}\right]\left[\begin{array}{c} \begin{array}{c} X_{w}\\ Y_{w} \end{array}\\ \begin{array}{c} Z_{w}\\ 1 \end{array} \end{array}\right]\left[\begin{array}{c} u\\ v\\ 1 \end{array}\right]=\left[\begin{array}{cc} \begin{array}{cc} a_{11} & a_{12}\\ a_{21} & a_{22}\\ r_{31} & r_{32} \end{array} & \begin{array}{cc} a_{13} & a_{14}\\ a_{23} & a_{24}\\ r_{33} & t_{3} \end{array} \end{array}\right]\left[\begin{array}{c} \begin{array}{c} X_{w}\\ Y_{w} \end{array}\\ \begin{array}{c} Z_{w}\\ 1 \end{array} \end{array}\right]$$

By ignoring the last row and using the concept of block matrix multiplication, we obtain the following Equation (5):

(5) $$\left[\begin{array}{cc} a_{11} & a_{12}\\ a_{21} & a_{22} \end{array}\right]\left[\begin{array}{c} X_{w}\\ Y_{w} \end{array}\right]+\left[\begin{array}{cc} a_{13} & a_{14}\\ a_{23} & a_{24} \end{array}\right]\left[\begin{array}{c} Z_{w}\\ 1 \end{array}\right]=\left[\begin{array}{cc} r_{31}u & r_{32}u\\ r_{31}v & r_{32}v \end{array}\right]\left[\begin{array}{c} X_{w}\\ Y_{w} \end{array}\right]+\left[\begin{array}{cc} r_{33}u & t_{3}u\\ r_{33}v & t_{3}v \end{array}\right]\left[\begin{array}{c} Z_{w}\\ 1 \end{array}\right]$$

Combining this matrix equation, we have the following Equation (6):

(6) $$\left[\begin{array}{cc} a_{11}-r_{31}u & a_{12}-r_{32}u\\ a_{21}-r_{31}v & a_{22}-r_{32}v \end{array}\right]\left[\begin{array}{c} X_{w}\\ Y_{w} \end{array}\right]=\left[\begin{array}{cc} r_{33}u-a_{13} & t_{3}u-a_{14}\\ r_{33}v-a_{23} & t_{3}v-a_{24} \end{array}\right]\left[\begin{array}{c} Z_{w}\\ 1 \end{array}\right]$$

It should be noted that, theoretically, there may be situations where it is impossible to have a reversible transformation from pixel coordinates $\left(u,v\right)$ to world coordinates. However, in practical scenarios, considering the constraints of camera parameters and other factors, we can assume that such a matrix is invertible and feasible. Thus, we have established a connection between $\left[\begin{array}{c} X_{w}\\ Y_{w} \end{array}\right]$ and $\left[\begin{array}{c} u\\ v \end{array}\right]$ as shown in Equation (7).

(7) $$\left[\begin{array}{c} X_{w}\\ Y_{w} \end{array}\right]={\left[\begin{array}{cc} a_{11}-r_{31}u & a_{12}-r_{32}u\\ a_{21}-r_{31}v & a_{22}-r_{32}v \end{array}\right]}^{-1}\left[\begin{array}{cc} r_{33}u-a_{13} & t_{3}u-a_{14}\\ r_{33}v-a_{23} & t_{3}v-a_{24} \end{array}\right]\left[\begin{array}{c} Z_{w}\\ 1 \end{array}\right]$$

Another prior piece of information is that, when using a relatively high-angle camera, even though the scale of the vessel may vary considerably during its journey, its edge shape typically remains relatively consistent. Therefore, the contour features of the vessel usually support our continuous tracking method, as described in the subsequent text.

Utilizing this information and combining the mapping relation vessel between ${\left[X_{w},Y_{w}\right]}^{T}$ and ${\left[u,v\right]}^{T}$, we can model the motion of the vessel under a monocular camera. Assuming that the edge shape of the vessel target remains relatively consistent, we consider the motion of the vessel target in the world coordinate system as rigid plane motion imaged in the pixel coordinate system.

**Figure 1:**
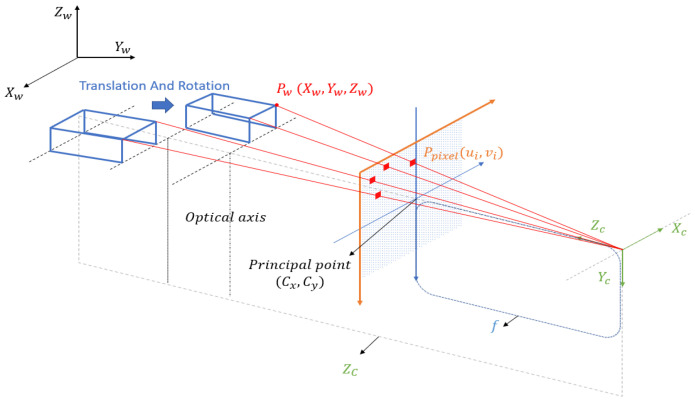
Imaging of the vessel’s motion.

Based on this assumption, the motion of the vessel in the world coordinate system can be represented as a combination of changes in heading angle and a translation vector in the pixel coordinate system. For a monocular camera, the motion of the vessel is imaged as shown in Figure 1.

The acquired trajectory composed of multiple frames of vessel feature points is further processed. The translation vector $T\left(t,t-1\right)$ from time t−1 to time t is computed using Equation (8), defined as the coordinate change of the mean of the feature points $P_{t}\left(u_{i},v_{i}\right)$ in the point cloud after removing outliers between consecutive frames. The change in the heading angle $R\left(t,t-1\right)$ from time t−1 to time t is calculated using Equation (9), which involves fitting the shape (e.g., linear) of the feature points between consecutive frames and obtaining the rotational component.

(8) $$T\left(t,t-1\right)=M\left(\sum_{i=1}^{N}\theta \left(P_{t}\left(u_{i},v_{i}\right)\right)\right)-M\left(\sum_{i=1}^{N}\theta \left(P_{t-1}\left(u_{i},v_{i}\right)\right)\right)$$

(9) $$R\left(t,t-1\right)=L\left(\theta \left(P_{t}\left(u,v\right)\right)\right)-L\left(\theta \left(P_{t-1}\left(u,v\right)\right)\right)$$

In this context, $\theta \left(\cdot \right)$ represents deleting warning values, $M\left(\cdot \right)$ stands for obtaining mean values, $L\left(\cdot \right)$ signifies overfitting of the shape, and $P_{t}\left(u_{i},v_{i}\right)$ denotes the pixel coordinates of point i.

For individual vessels, the aforementioned formula is applicable. However, when processing the trajectories of multiple vessels simultaneously, the handling of the acquired tracking points differs. Initially, it necessitates the application of a clustering algorithm to these tracking points in each frame to ascertain the cluster centers of multiple vessels. The alteration in the cluster centers is then used to represent their trajectories. To capitalize on the advantage of tracking points encompassing the contours of the vessels, a Principal Component Analysis (PCA) is conducted on these points to determine their principal axis direction, which serves as the course direction. This approach facilitates the simultaneous processing of data from multiple vessels, without further elaborating on the formula.

### 2.2. Motion Estimation Method

Drawing inspiration from the ideas and methods of Co-tracker, the following definitions are made for the concepts used in tracking vessel feature point clouds. The long-term video sequence is regarded as being composed of T frames of RGB images, denoted as follows:

$$V={\left(I_{t}\right)}_{t=1}^{T},\;I_{t}\in R^{3\times H\times W}.$$

In the video to be tracked, any number of pixels is denoted as follows:

$$P_{t}^{i}=(x_{t}^{i},y_{t}^{i}),\;t=u,\ldots ,T;i=1,\ldots ,N.$$

where u represents the initial time for tracking initiation. To represent the visual status of each point and indicate whether tracking has failed, the visual status is defined as follows:

$$v_{t}^{i}=\left\{0,1\right\}$$

where 1 indicates that the pixel point can be tracked, and 0 indicates that it is occluded or tracking has failed. Therefore, the entire tracking problem for the vessel’s feature point cloud is defined as estimating the positions of the N tracking points specified in the first frame in the subsequent T−u frames.

To optimize the estimation of the tracking point trajectories, Co-tracker’s approach is based on the idea that estimating the overall motion of an object should not ignore the constraining effect of different points on the object. Just as points on the surface of an object are constrained by nearby points, it is necessary to obtain global features of an object or, in other words, to obtain appearance features $\phi \left(I_{t}\right)$ corresponding to each frame. These appearance features are then down-sampled to initialize the tracking features $Q_{t}^{i}\in R^{d}$ upon which the tracking points depend. Subsequently, the position estimation of the tracking points is iteratively optimized using these tracking features.

To consider the constraints between related points, they introduce related feature vectors $C_{t}^{i}\in R^{S}$. These vectors are obtained by stacking the inner product of the image features $\phi \left(I_{t};s\right)$ normalized by the estimated positions with the tracking features $Q_{t}^{i}$, as expressed in Equation (10):

(10) $$C_{t}^{i}=<Q_{t}^{i},\phi \left(I_{t};s\right)\left[\frac{{\hat{P}}_{t}^{i}}{ks+\delta }\right]>$$

Subsequently, they apply Transformer theory to iteratively optimize N tracking points in each frame for M iterations to obtain the most accurate estimates. Each iteration’s input includes the current position estimates ${\hat{P}}_{t}^{i}$, the activation of the initial visual states $\mathrm{logit}\left({\hat{v}}_{t}^{i}\right)$, tracking features $Q_{t}^{i}$, related features $C_{t}^{i}$, and motion encoding ${\hat{P}}_{t}^{i}-{\hat{P}}_{1}^{i}$, where γ represents the sine positional encoding, as shown in Equation (11):

(11) $$G_{t}^{i}=({\hat{P}}_{t}^{i},logit\left({\hat{v}}_{t}^{i}\right),Q_{t}^{i},C_{t}^{i},\gamma \left({\hat{P}}_{t}^{i}-{\hat{P}}_{1}^{i}\right)$$

Using $\varphi \left(\cdot \right)$, which is the output after applying the Transformer, they update the estimates of the positions and tracking features, as shown in Equation (12):

(12) $$O\left({\hat{P}}^{\left(m+1\right)},Q^{\left(m+1\right)}\right)=\varphi \left(G\left({\hat{P}}^{\left(m\right)},{\hat{v}}^{\left(0\right)},Q^{\left(m\right)}\right)\right)$$

Throughout the M iterations, when the defined losses (e.g., cross-entropy loss for visible states and loss for tracking point estimation, not discussed in detail here) meet the requirements, the estimation of the tracking point positions is achieved.

**Figure 2:**
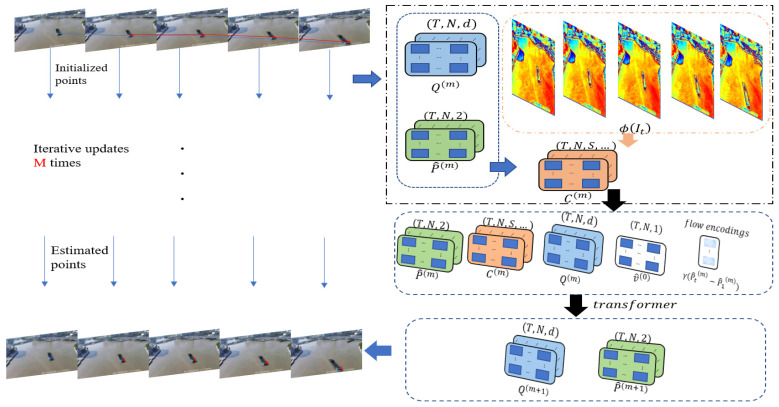
The framework for tracking the motion trajectories of multiple feature points.

The main workflow of the multi-feature point motion trajectory method described above is as shown in Figure 2:

In practical engineering applications for tracking multiple feature point clusters on vessels, there is a need to track as many feature points as possible to improve accuracy while ensuring that feature points are not overly dense to save resources and increase speed. This led to the development of an efficient multi-feature point tracking method by combining the characteristics of Co-tracker to obtain related feature points. To track vessels efficiently, instance segmentation is used to acquire masks as constraints.

However, the effectiveness of mainstream instance segmentation methods is often compromised when segmenting ship instances against complex ocean backgrounds. This challenge primarily arises from three aspects:

- Complex Backgrounds:

The intricate nature of oceanic settings, characterized by waves, light reflections, and water surface textures, creates visual similarities with the ship instances [25]. This similarity hinders the segmentation algorithm’s ability to distinguish the ships from their background, thereby impacting the deep learning model’s performance and its precision in accurately locating and generating semantic masks for each instance.

- Small Object Recognition:

The limited pixel representation of small objects in images results in insufficient feature information, making them challenging for standard deep learning models to recognize effectively. Small objects often blend into the background, further complicating recognition. Employing Transformer methods [26] in Co-tracker classes can notably enhance the recognition of these small objects.

- Blurred Boundaries:

Boundary blurring in segmentation models can be attributed to either the inherent resolution limitations of the model or its inability to effectively process complex boundaries [27]. This limitation leads to the algorithm’s inadequate detail resolution, meaning the segmentation cannot accurately delineate the contours of an object.

Addressing the issue of accurate edge detection in segmentation models requires integrating specialized algorithms. However, this approach encounters significant challenges in water surface scenarios. Traditional edge detection algorithms are particularly sensitive to noise elements like reflections and ripples, often leading to frequent erroneous detections. Additionally, the segmentation of ship bottom lines is adversely affected by water surface fluctuations, especially during the movement of ships. Another hurdle is the dependency of some algorithms on fixed thresholds, which proves less effective in dynamic environments and necessitates manual adjustments to suit varying conditions. While classic convolutional neural network (CNN)-based methods, such as the Richer Convolutional Features (RCF) model [28] used in this research, offer strong robustness, they also face substantial computational demands and issues like edge blurring when processing entire images. Currently, these challenges remain without an ideal solution.

This paper adopts the instance segmentation results to obtain the approximate position of the contour and then utilizes convolutional-network-based edge detection to obtain refined contour shapes. This approach strikes a balance between the time and precision requirements for obtaining the actual contours of vessel targets.

The specific method involves obtaining the bounding boxes from instance segmentation and then performing super-resolution interpolation or direct edge detection within these boxes. Some erosion operations are applied to remove excess interference. Next, the mask obtained from instance segmentation is fused with the edges obtained from edge detection. This fusion typically involves simple intersection and union operations, which are beneficial in most cases.

By using masks as constraints, this approach not only facilitates the computation of related features but also conserves computational resources during training. However, in the case of long-time sequences and dense point tracking, it can be computationally intensive during inference. In Co-tracker, a sliding window approach is used to slice long time sequences and then utilize the estimates from the previous window as input to continue tracking a fixed group of target points in the next window. While this approach results in highly accurate feature points, it may not be ideal for tracking, for example, tiny vibrations in bridge model regions. This is because the essence of this tracking method is the iterative optimization of estimated point positions, which inherently introduces uncertainty. When combined with high-frequency tiny vibrations that are difficult to measure directly in bridge models, the tracking becomes exceptionally challenging.

**Figure 3:**
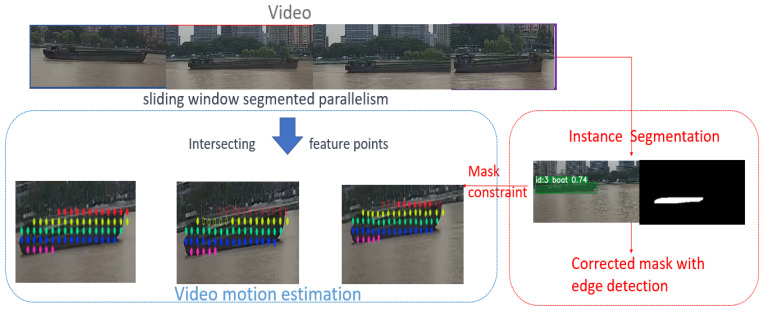
Maritime vessel trajectory acquisition framework.

However, when it comes to obtaining the trajectories of vessels, this method can leverage its maximum advantage, as depicted in the framework proposed in Figure 3 below:

Because modeling maritime vessel motion relies on the statistical properties of these tracking points, there is no need to use a sliding window approach, as in the original work, where the previous time step’s output serves as the input for the next one. It is possible to divide the video into smaller segments without concern for the consistency of tracking points, enabling the parallel acquisition of these tracking point clusters, and ultimately achieving efficient inference through timestamp alignment.

In our methodology, video footage is segmented into smaller units, approximately 15 frames per segment, facilitating detailed analysis. At the onset of each segment, instance segmentation is employed, with the initial frame’s segmentation mask serving as a pivotal constraint for tracking maritime vessel movement. This targeted approach enables the efficient and precise extraction of vessel feature points. Utilizing a substantial number of these tracking points, our method facilitates the computation of translation and rotation, thereby acquiring consistent and continuous vessel trajectories. This process potentially outperforms the direct usage of the Co-tracker method in terms of efficiency.

**Figure 4:**
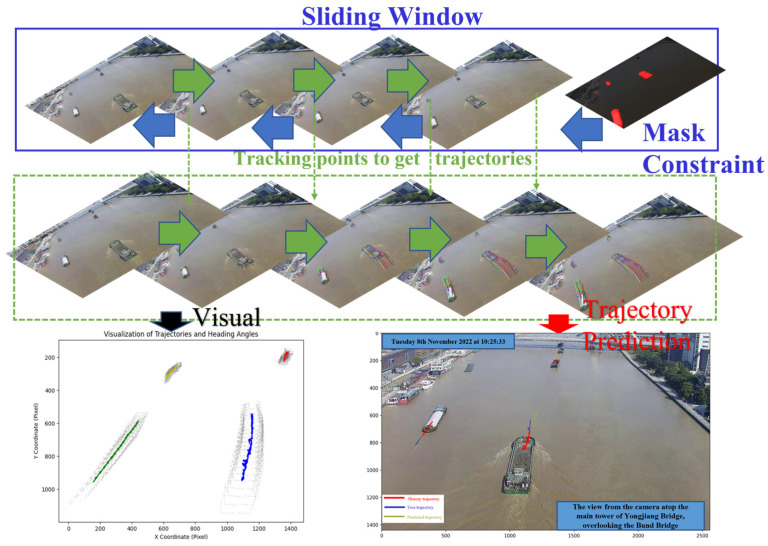
Technique for the acquisition and prediction of vessel trajectories.

The scalability of our approach in densely trafficked maritime areas is systematically illustrated in Figure 4. This depiction showcases the method’s capability in tracking and predicting vessel trajectories in scenarios involving multiple objects over extended periods. Utilizing a sliding window technique, each video segment’s initial frame acts as a foundational mask constraint to acquire critical trajectory tracking points. These points are then employed in the prediction and visualization of vessel paths, highlighting the method’s practicality in both real-time tracking and future path forecasting. Figure 4 visually delineates this methodology: the upper section depicts the sliding window segmentation, the central part illustrates the trajectory tracking process, and the lower section contrasts predicted trajectories with actual vessel movements.

To address the complexities of multi-vessel scenarios, we have developed specialized clustering and data processing algorithms. These algorithms leverage the movement of clustering centers to calculate displacement and use Principal Component Analysis (PCA) to ascertain the main axis direction. A key aspect of these algorithms is their focus on processing a long sequence of tracking points, essentially handling a four-dimensional array. This computational approach is designed to meet real-time processing requirements, efficiently managing the volume of data typical in extended maritime surveillance.

However, we have identified a limitation within our current approach: as vessels recede and diminish in size, the PCA-based main axis direction may undergo abrupt alterations. This challenge becomes particularly pronounced in the context of real-time analysis, where the dynamic nature of maritime environments necessitates the rapid adaptation of the algorithms. These observations underscore the necessity for more advanced algorithmic solutions capable of accommodating dynamic changes in vessel appearance and movement, while maintaining the real-time processing capabilities essential for effective maritime surveillance and navigation safety.

## 3. Trajectory Prediction

In the Ningbo Sanjiangkou area, maritime transportation is highly accessible, and it is common to see fleets of vessels sailing together or anchoring on the side, resulting in a multi-channel, two-way maritime transportation scenario. This creates a complex navigation environment for vessels within the region, with intricate navigation relation vessels.

Faced with complex maritime navigation scenarios, vessel navigation involves information exchange among vessels. When predicting vessel trajectories in such situations, it is important to consider the spatiotemporal interactions between vessels. Inspired by Huang [24] and colleagues’ work on pedestrian trajectory prediction methods, a simplified version of a spatiotemporal graph attention neural network was constructed.

**Figure 5:**
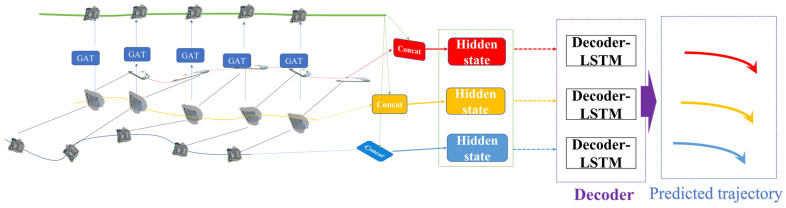
A simplified version of the spatiotemporal graph attention neural network framework.

As shown in Figure 5, the first step involves using LSTM methods to capture the temporal correlations in the historical trajectory data of each vessel. To achieve this, the relative positions of each vessel in each frame with respect to the previous frame are obtained as inputs, as shown in Equation (13):

(13) $$\delta u_{i}^{t}=u_{i}^{t}-u_{i}^{t-1},\delta v_{i}^{t}=v_{i}^{t}-v_{i}^{t-1}$$

The relative position encoding, along with the embedding weights $W_{e}$, transformed based on distance, are embedded into a fixed-length vector $e_{i}^{t}$ before being input into the LSTM to capture time-related features, as shown in Equation (14):

(14) $$m_{i}^{t}=LSTM\left(m_{i}^{t-1},e_{i}^{t},W_{m},\right),e_{i}^{t}=\varphi (\delta u_{i}^{t},\delta v_{i}^{t},W_{e})$$

Furthermore, a graph attention neural network is employed to capture the spatial correlations among individual vessels within the vessel group at each time step. The spatial correlations at each time step are then input into the LSTM to obtain the spatiotemporal correlations related to spatial relation vessels. In this context, a graph attention neural network is utilized to assign different weights to different vessel nodes, aggregating information from neighboring nodes to obtain spatial correlations.

By inputting the obtained temporal information features $m_{i}^{t}$ into the graph attention layer, the normalized attention coefficients $\alpha_{ij}^{t}$ between nodes are calculated. Subsequently, the results from each node, weighted by the attention coefficients, are aggregated and activated to produce spatiotemporal interaction features that take into account spatial interactions, as shown in Equation (15):

(15) $${\hat{m}}_{i}^{t}=\sigma \left(\sum_{j\in N_{i}}\alpha_{ij}^{t}Wm_{j}^{t}\right)$$

**Figure 6:**
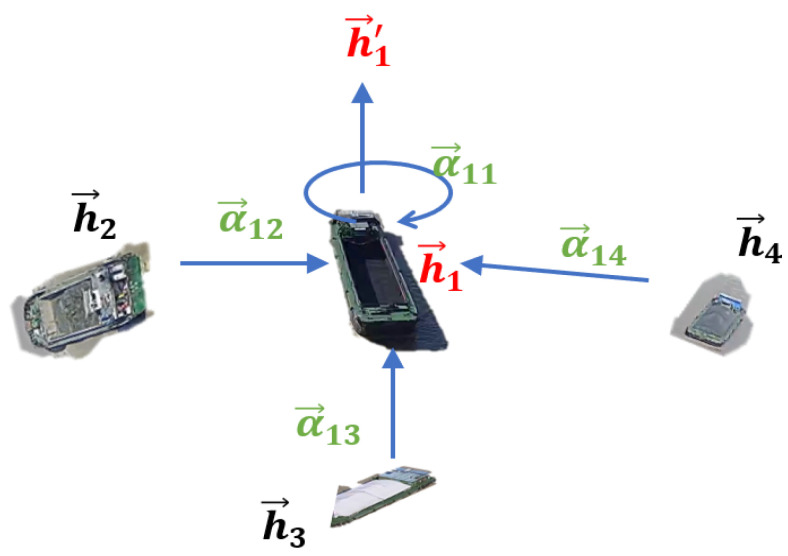
Graph attention layer.

As shown in Figure 6, a graph is used to depict the interactions between vessels. The spatial interactions among multiple vessels within a time window are represented as a graph composed of multiple nodes and edges. The GAT (Graph Attention Network) in Figure 6 is implemented by stacking multiple layers of graph attention layers, as illustrated in Figure 6 (the code uses two layers). This allows for the encoding and decoding of spatiotemporal correlations, enabling the construction of a spatiotemporal trajectory prediction model for vessel trajectory prediction.

In terms of system architecture, the framework comprises two main components deployed on dual servers. The trajectory acquisition method, Co-tracker, fundamentally involves tracking dense feature points over extended sequences. Once these tracking points are obtained, post-processing is conducted to acquire the trajectories and other feature information. The trajectory prediction method, on the other hand, utilizes the obtained trajectories to infer future paths. This framework, comprising these two elements, is characterized by its low complexity, moderate integration, and ease of maintenance.

The computational demands of this system are primarily dictated by the Co-tracker and RCF components, with parameter counts of 24,149,859 and 14,803,781, respectively. In contrast, our constructed trajectory prediction module, with a total parameter count of 44,630, demands significantly less computational power compared to current large language models.

In the proposed methodological framework of this study, the primary consumption of GPU memory is not attributed to the model loading processes. Instead, the predominant portion of the GPU memory is utilized by the Co-tracker, particularly due to the conversion of videos into tensors. The methodology employs the parallel processing of multiple short video segments, which, under similar computational loads, allows multi-threading techniques to significantly accelerate the acquisition of tracking points. However, this approach still entails substantial memory consumption, especially for real-time processing.

Among all the models requiring training in our framework, YOLOv8n-seg and STGAT are the primary focus. The other methods, owing to the general applicability of the models we have employed, necessitate only minimal fine-tuning to adapt to various scenarios. Moreover, these models boast the advantage of having relatively smaller parameter sizes and rapid inference capabilities. On an RTX 4080 GPU, these models are capable of achieving efficient parallel processing.

For a segmented image with a resolution of 1920 × 1080, the inference speeds of various components on an RTX 4080 are shown in Table 2 below:

However, a significant limitation of these methods lies in the demands for GPU memory and server memory. During the parallel processing of trajectory recognition algorithms, the memory consumption is approximately 32 GB, with around 15.5 GB of GPU memory usage.

Regarding data quality and model generalization, our research focuses mainly on the trajectory prediction model and the Yolov8n-seg segmentation model. Due to the strong generalization capabilities of Co-tracker and RCF, the requirements for data quality can be moderately reduced. Notably, the Co-tracker model, which significantly differs from typical self-supervised learning methods, shows superior universality in learning the interrelationship between motion feature points and optical flow, especially in predicting new trajectory points. In varying maritime conditions, the Co-tracker model effectively captures the interactions among feature points, exhibiting impressive adaptability. However, it is essential to note that front-end models like YOLOv8n-seg or RCF might be affected by environmental factors, impacting mask acquisition and the number of effective tracking points. While the impact is minimal in well-calibrated and favorable maritime settings, the performance of our system under extreme sea conditions remains to be tested.

## 4. Case Analysis

### 4.1. Project Background

**Figure 7:**
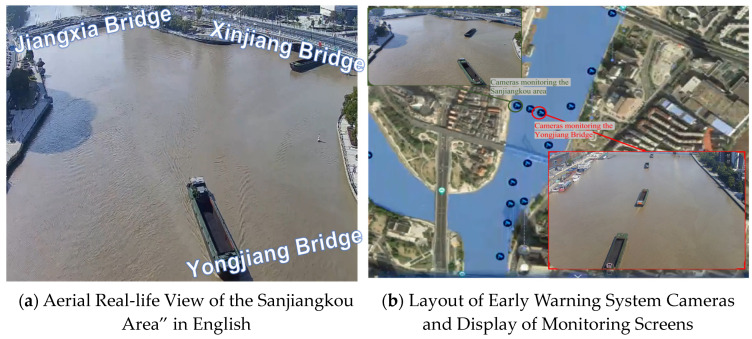
Sensor deployment in the Sanjiangkou area.

As shown in Figure 7, the Sanjiangkou area in Ningbo serves as a critical hub for waterborne transportation. It is characterized by the convergence of three rivers, a dense network of waterways, and numerous bridges spanning these water channels. However, some of these cross-river bridges, due to their age, have limited navigational clearance, leading to occasional bridge collisions by vessels, thus posing a high risk to maritime safety. In this region, Xia [9] have established a comprehensive Bridge Collision Prevention and Early Warning System, which integrates multiple types of equipment, various monitoring methods, and data from multiple sources. Notably, 32 cameras have been strategically placed at the confluence of the three rivers to provide coverage for this area. In the ensuing case study, the primary images employed are sourced from Camera No. 2018 and Camera No. 2019, as depicted in Figure 7b. The camera locations are strategically positioned to offer a panoramic view of the Sanjiangkou area.

In the face of complex maritime traffic conditions in the Sanjiangkou area, the core modules that enable proactive warning functionality are the trajectory acquisition and trajectory prediction modules. The following two cases will focus on trajectory acquisition from video sequences for vessel targets and trajectory prediction based on historical vessel tracks within the navigational area.

### 4.2. Metrics for Evaluating the Effectiveness of Trajectory Recognition

To establish a comprehensive performance index for ship trajectory recognition that quantifies the accuracy and stability of the model, we consider the following metrics:

1. Trajectory Modeling Accuracy (TMA):

This metric quantifies the average deviation between the predicted trajectory and the true trajectory. Given a predicted trajectory $\left(P=\left\{p_{1},p_{2},\ldots ,p_{n}\right\}\right)$ and a true trajectory $\left(T=\left\{t_{1},t_{2},\ldots ,t_{n}\right\}\right)$, where $p_{i}$ and $t_{i}$ are the predicted and true positions at the i-th frame, respectively, the TMA is calculated as follows:

(16) $$TMA=\frac{1}{n}\overset{n}{\underset{i=1}{\sum }}d\left(p_{i},t_{i}\right)$$

where $d\left(p_{i},t_{i}\right)$ denotes the Euclidean distance between the predicted and actual positions. A lower TMA indicates higher precision in trajectory prediction.

2. Trajectory Stability (TS):

$$\frac{Std\;Acceleration}{Mean\;Acceleration}*Mean\;Directional\;Change:$$

This calculation combines the acceleration variability (standard deviation to mean ratio) with mean directional change. High values indicate substantial acceleration changes and significant directional shifts, suggesting an erratic trajectory.

The formula for $\frac{\mathrm{Std}\;Acceleration}{Mean\;Acceleration}$ is as follows:

(17) $$\frac{Std\;Acceleration}{Mean\;Acceleration}=\frac{\sqrt{\frac{1}{N}\sum_{i=1}^{N}{\left(A_{i}-\frac{1}{N}\sum_{i=1}^{N}A_{i}\right)}^{2}}}{\frac{1}{N}\sum_{i=1}^{N}A_{i}}$$

Let $A_{i}$ represent the acceleration at the i-th observation and N be the total number of observations.

The mean directional change quantifies the degree of directional change between consecutive points on the trajectory. Lower directional change values indicate greater directional stability. The formula for Mean Directional Change is as follows:

(18) $$Mean\;Directional\;Change=\frac{1}{N-1}\overset{N-1}{\underset{i=1}{\sum }}abs\left(\theta_{i}-\theta_{i-1}\right)$$

where $\theta_{i}$ is the direction angle at the i-th point.

Mean curvature represents the average curvature of the entire trajectory, indicating its overall level of curvature. Lower Mean Curvature values suggest a smoother trajectory, closer to a straight line. The formula for Mean Curvature is as follows:

(19) $$Mean\;Curvature=\frac{1}{N}\overset{N}{\underset{i=1}{\sum }}abs\left(\kappa_{i}\right)$$

where N is the number of trajectory points and $\kappa_{i}$ is the curvature at the i-th point. The TS metric combines these three indicators:

(20) $$TS=\frac{Std\;Acceleration}{Mean\;Acceleration}*Mean\;Directional\;Change+Mean\;Curvature$$

When these three components are combined to compute the Trajectory Stability metric, a smaller TS value indicates that the trajectory is stable in terms of velocity, direction, and curvature. Conversely, a larger TS value suggests significant fluctuations or changes in these aspects. This comprehensive assessment provides valuable insights into the overall stability characteristics of the trajectory.

3. Fréchet Distance (FD):

The Fréchet Distance is a measure of the similarity between two curves, accounting for both the position and ordering of points along the trajectories.

Combining these metrics, we propose the Trajectory Recognition Comprehensive Performance Index (TRCPI), a metric that integrates trajectory accuracy, stability, and shape similarity, defined as follows:

(21) $$TRCPI=\alpha \cdot \mathrm{TMA}-\beta \cdot \mathrm{TS}+\gamma \cdot FD\left(P,T\right)$$

where $FD\left(P,T\right)$ represents the Fréchet Distance between the predicted and true trajectories, reflecting the geometric similarity and considering the sequence of locations. The factors α, β, and γ are weighting coefficients that adjust the relative importance of the three metrics within the overall index.

The TRCPI metric synthesizes trajectory precision, stability, and path similarity, providing a multidimensional quantitative measure for evaluating ship trajectory recognition methods. By adjusting the weighting coefficients, the TRCPI can be tailored to emphasize different performance requirements according to the specific needs of the application scenario.

### 4.3. Real Vessel Experiment

**Figure 8:**
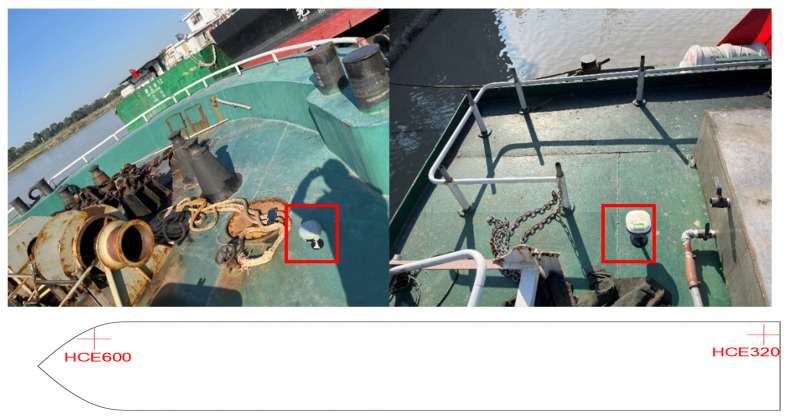
Experimental ship GPS equipment layout.

**Figure 9:**
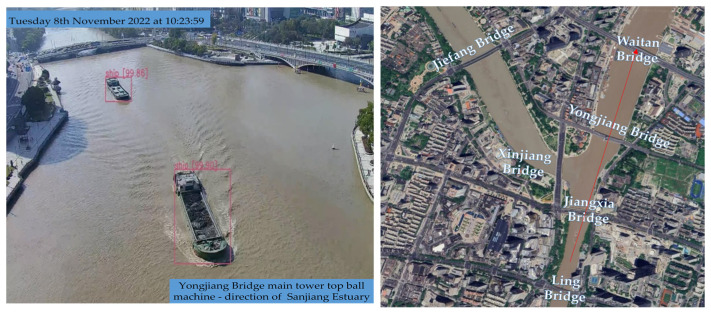
Driving route and the pre-established test results.

To validate the efficacy of the proposed method and the functionality of the active early warning system in the Sanjiangkou area, a 2-day ship test was conducted in the same location. Control experiments were executed using the YOLOv8 model integrated with Bytetrack, the framework presented in this study, and GPS to obtain the ship trajectory. GPS devices were strategically installed at the bow and stern of the ship, as illustrated in Figure 8 below:

The primary experimental route extends from Jiangxia Bridge to Yongjiang Bridge, encompassing the Sanjiang Estuary area. Both the driving route and the pre-established test results are illustrated in Figure 9.

**Figure 10:**
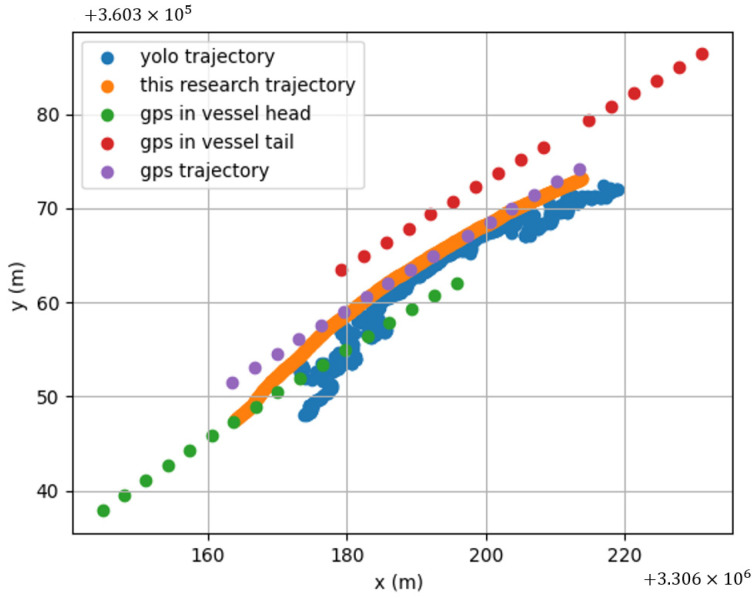
Each method obtains the trajectory.

We analyzed the video and GPS data of the ship’s journey from Jiangxia Bridge to Yongjiang Bridge and converted the pixel track in the video to the actual trajectory, as depicted in Figure 10 below.

Given the challenges in accurately calibrating the ball machine in real engineering scenarios within the Sanjiangkou area, there exists a certain margin of error in the homography matrix. This discrepancy introduces a deviation between the trajectory derived from video images and the actual trajectory. In Figure 10, the red and green scatter points represent GPS data from the bow and stern, respectively, while the purple trajectory signifies the ship’s center, obtained through mean value calculation. The inherent scattering of the trajectory is attributed to the 1 Hz GPS data transmission frequency. Notably, the trajectory obtained by the framework proposed in this study exhibits greater stability and accuracy compared to that obtained by the YOLO model. Furthermore, the video image method produces a trajectory with higher resolution than GPS, facilitating the extraction of deep features for improved track prediction. This attribute aligns with the objectives of the proposed framework, showcasing its potential for advancements in this field.

**Figure 11:**
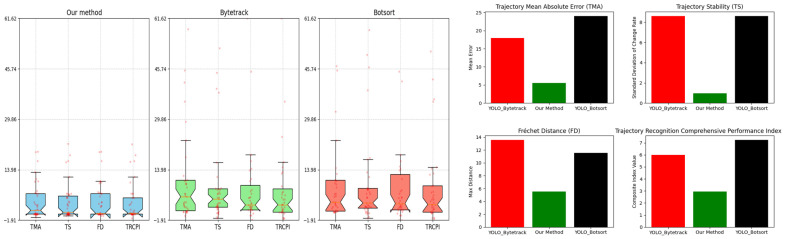
Comparison of metrics between the three methods.

To quantitatively evaluate the performance of trajectory recognition, we will employ the metrics introduced in Section 4.2. We will compute the Trajectory Recognition Comprehensive Performance Index (TRCPI), setting the parameters as follows: α=0.3, β=0.4, and γ=0.3. To mitigate accidental factors and minimize the impact of camera distortion at the track’s ends, we conducted an experimental evaluation of the effectiveness of the indicators proposed in this study. We extracted 39 video segments from recordings made by three cameras. For each video, we manually identified the true center point of the ship every ten frames. The evaluation employed the integration of YOLO with Bytetrack, YOLO with Botsort, and the method introduced in this paper. The statistical mean and box plot representations of these indicators are depicted in Figure 11.

The results indicate that our method holds a distinct advantage in terms of stability, as shown by its lower spread in the box plots compared to the other methods. Additionally, it demonstrates superior performance in accuracy and trajectory similarity, which is evidenced by the tighter clustering of data points and the more favorable indices in the corresponding charts. Our approach significantly outperforms the other methods, reflecting its robustness and reliability in tracking applications.

### 4.4. Holistic Case Application

**Figure 12:**
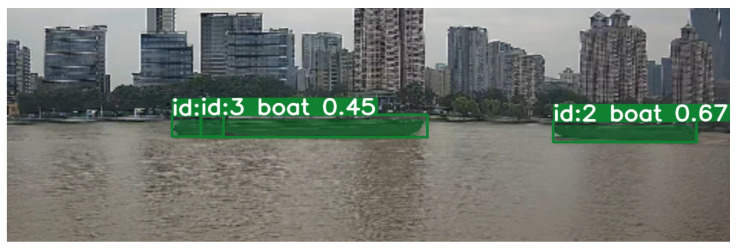
Vessel false detection condition.

Faced with various situations, most projects employing the Yolo object detection algorithm combined with the DeepSORT tracking algorithm or those utilizing vessel trajectory methods obtained through instance segmentation have inherent limitations. These methods essentially involve object detection across multiple frames, acquiring their bounding boxes or instance masks, and then employing techniques such as appearance features or other methods to associate objects detected in consecutive frames, achieving tracking effects. As a result, the quality of tracking heavily relies on the accuracy and stability of the initial detection, and bounding boxes do not always precisely align with the object’s edges. Using the trajectory of a single point on the bounding box as the vessel’s trajectory can introduce considerable errors. While this drawback can be mitigated by adjusting the sensor’s viewing angle, it is not always feasible to have ideal image data from a better perspective. Alternatively, the direct utilization of instance segmentation to acquire image masks for tracking front and rear frame masks can also introduce instability. In certain poor viewing angles, it may even lead to false positives or missed detections, as illustrated in Figure 12. This, without a doubt, poses a catastrophic challenge for trajectory recognition.

**Figure 13:**
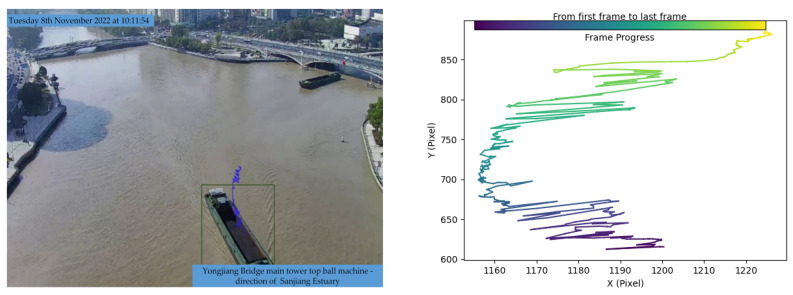
Obtaining vessel trajectories based on bounding boxes

**Figure 14:**
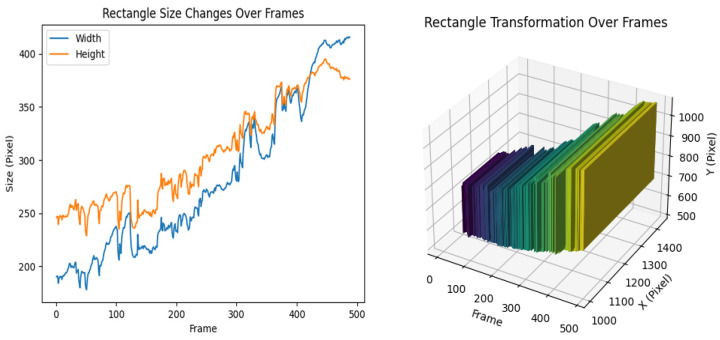
Bounding box variations.

**Figure 15:**
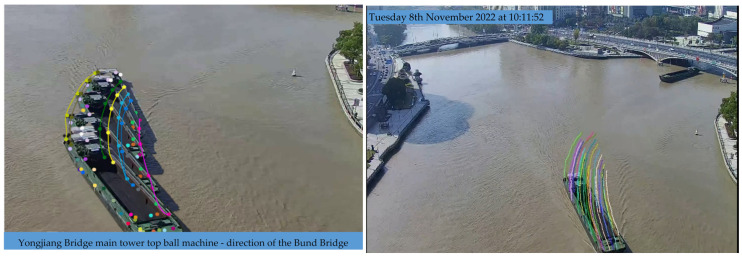
Multi-feature point cluster tracking method.

Even in the case of a favorable overhead view with excellent recognition results, theoretically, the center point movements of the bounding boxes obtained using the Yolo algorithm could be used as vessel trajectories. For the scenario depicted in Figure 13, vessel trajectories were obtained by analyzing the changes in bounding boxes using the Yolo algorithm combined with the Bytetrack algorithm for a 484-frame video sequence. The variations in the bounding boxes are illustrated in Figure 14. As the timeline progresses, the bounding boxes often exhibit abnormal peaks within a certain time period, and there are cases where the width suddenly exceeds the height. It is evident that the trajectory recognition for the scenario shown in Figure 15 has been severely affected. For a video sequence consisting of only 484 frames, the instability of the bounding boxes obtained by the Yolo algorithm results in highly unstable trajectory recognition. This also explains why the combination of the Yolo object detection algorithm and the DeepSORT tracking algorithm exhibited subpar performance in the experiments conducted in Section 4.3.

Using the improved long-time sequence multi-feature point cluster tracking motion estimation method, as shown in the figure below, there are two approaches based on the length of the time sequence. The first approach is for relatively short time sequences. When computational resources are sufficient, the entire time sequence can be directly input, resulting in a continuous array of multi-frame feature point clusters, as shown in Figure 15. These can be visualized as point trajectories, as shown in Figure 15.

**Figure 16:**
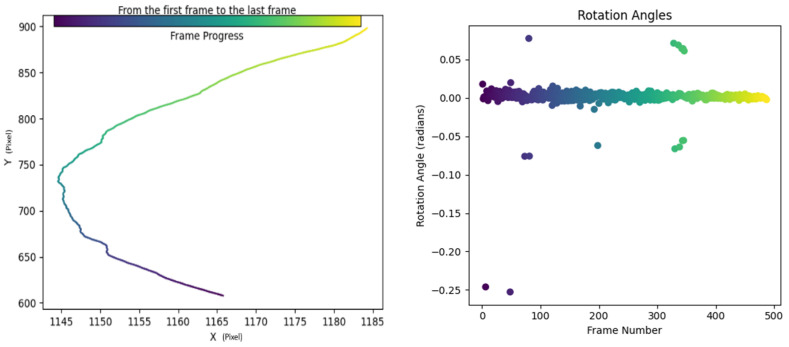
Long-term vessel trajectories and heading angles.

However, when the time sequence is too long, it is divided into slices. In this case, the focus shifts from pursuing the consistency of tracked points to the parallel processing of multiple batches of sliced videos, each input into the model to obtain multiple sets of tracked points. The statistical characteristics of these tracked points are then directly obtained, as shown in Figure 16.

**Figure 17:**
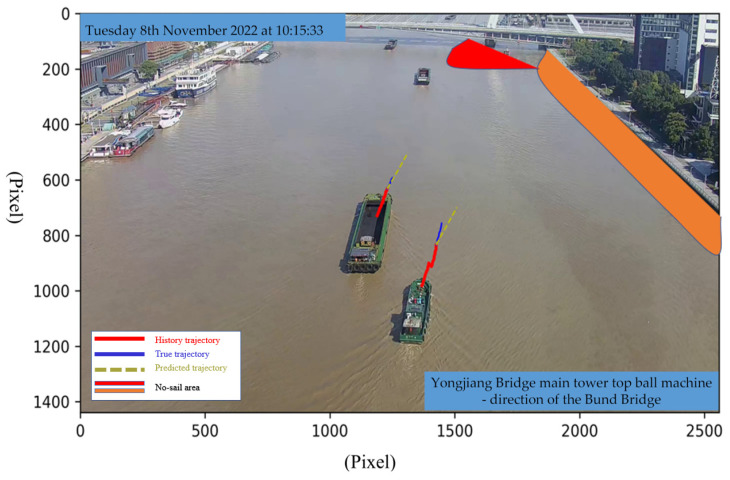
Predictive and alert illustration diagram.

Upon acquiring vessel trajectories, they are input into the trajectory prediction model, yielding results as illustrated in the accompanying figure. In Figure 17, the predicted trajectories are represented in yellow, the actual trajectories in blue, and the historical trajectories in red. Notably, the no-sailing zones are also depicted. We will exemplify the method of collision detection between vessels and between vessels and bridges as follows:

- Vessel-to-Vessel Collision Detection:

In scenarios involving multi-target vessel navigation, we cluster the series of tracking points to generate scatter plots for multiple vessels. During the prediction phase, we calculate the Intersection Over Union (IOU) between the scatter plots of two vessels. This approach allows for collision predictions with a certain degree of safety redundancy.

- Vessel-to-Bridge Collision Alert:

Similarly, during the prediction phase, the IOU between the vessel and no-sailing zones (such as bridges) is calculated. This method is used to predict potential collisions between vessels and bridges.

This translation and refinement emphasize the academic rigor and technical sophistication of the trajectory prediction model and collision detection methodology. The approach focuses on the integration of advanced analytical techniques to enhance maritime safety.

## 5. Conclusions

This study aims to enhance the method for vessel trajectory recognition involving multiple features in wide areas, multiple objectives, long durations, and complex environments, addressing the limitations of traditional approaches. The following are the main contributions and conclusions of this study:

1. The improved method for vessel trajectory recognition with long-term, multi-feature point sequences demonstrates outstanding accuracy and efficiency. It exhibits clear advantages over traditional methods. This method is expected to be a major direction for future research because it not only provides high-precision trajectory data but is also applicable to various scenarios without the need for extensive data training. It can be run simply by combining the SAM model with silhouette masks for feature-point-constrained inputs. However, it should be noted that this method requires relatively high computational resources, which may necessitate further research in hardware and performance optimization.
2. This paper makes an assumption regarding the world coordinates $Z_{w}$ of vessels within a relatively small area, simplifying the actual movement of vessels within that region to rigid body motion on a plane. It utilizes statistical methods to obtain vessel motion parameters from the tracked multiple feature points. This assumption and method offer an effective approach to describe complex vessel movements and are expected to find broad applications in areas with developed maritime transportation, such as waterways.
3. The trajectory prediction method in this paper takes full account of the spatiotemporal correlations in vessel trajectories, making it particularly suitable for well-established maritime regions where vessels often travel in formations. While the trajectory prediction method in this paper has made significant advancements, there is still room for improvement. The current limitations in data quality and quantity constrain the optimization of this method. Future work can focus on obtaining higher-quality data and further enhancing the prediction model to improve its performance and applicability.

In general, the research presented in this paper offers valuable novel methodologies and concepts for the field of vessel trajectory recognition and prediction. These pioneering approaches are anticipated to play a pivotal role in vessel traffic management and maritime navigation safety in the future, while also providing guidance and inspiration for subsequent investigations.

## Figures and Tables

**Table 1:** The main active vessel-bridge collision prevention sensor devices.

Method	Synthetic Aperture Radar	Camera	Automatic Recognition System
Weather adaptability	Strong universality, suitable for a variety of environments (white, day, sunny, rain), high precision [10]	Dependent on lighting, best performance in clear weather	Unaffected
Continuous monitoring capability	Strong (All time)	Dependent on lighting, requires auxiliary equipment at night	Unaffected
Sea Condition Adaptability	Strong	Moderate	Strong
Accuracy	Strong	Moderate	Instability
Cost	High	Low	The construction cost is high, the use cost is low [11]
Main advantages	High resolution, wide coverage	Rich image features	Provides detailed ship information
Main disadvantages	Complex operation	Limited viewing angle and lighting	Reporting frequency and accuracy are limited

**Table 2:** The average inference speeds of various components on an RTX 4080.

Motion Estimation Module—Co-Tracker	Edge Detection Module—RCF	Segmentation Module		Prediction Module
		Yolov8n-seg	SAM	
12 s for processing 250 frames	30 frames per second (FPS)	8 ms per frame	0.452 s per frame	54 ms per frame on GPU

## Data Availability

The data presented in this study are available on request from the corresponding author. The data are not publicly available due to confidentiality of some project data with time-limited factors.
